# Immuno-biosensor for Detection of CD20-Positive Cells Using Surface Plasmon Resonance

**DOI:** 10.15171/apb.2017.023

**Published:** 2017-06-30

**Authors:** Dariush Shanehbandi, Jafar Majidi, Tohid Kazemi, Behzad Baradaran, Leili Aghebati-Maleki, Farzaneh Fathi, Jafar Ezzati Nazhad Dolatabadi

**Affiliations:** ^1^Immunology Research Center, Tabriz University of Medical Sciences, Tabriz, Iran.; ^2^Student Research Committee, Tabriz University of Medical Sciences, Tabriz, Iran.; ^3^Research Center for Pharmaceutical Nanotechnology, Tabriz University of Medical Sciences, Tabriz, Iran.

**Keywords:** CD20, Surface Plasmon Resonance, Immobilization, Staphylococcus aureus protein A, 11-mercaptoundecanoic acid

## Abstract

***Purpose:*** Surface plasmon resonance (SPR) sensing confers a real-time assessment of molecular interactions between biomolecules and their ligands. This approach is highly sensitive and reproducible and could be employed to confirm the successful binding of drugs to cell surface targets. The specific affinity of monoclonal antibodies (MAb) for their target antigens is being utilized for development of immuno-sensors and therapeutic agents. CD20 is a surface protein of B lymphocytes which has been widely employed for immuno-targeting of B-cell related disorders. In the present study, binding ability of an anti-CD20 MAb to surface antigens of intact target cells was investigated by SPR technique.

***Methods:*** Two distinct strategies were used for immobilization of the anti-CD20 MAb onto gold (Au) chips. MUA (11-mercaptoundecanoic acid) and Staphylococcus aureus protein A (SpA) were the two systems used for this purpose. A suspension of CD20-positive Raji cells was injected in the analyte phase and the resulting interactions were analyzed and compared to those of MOLT-4 cell line as CD20-negative control.

***Results:*** Efficient binding of anti-CD20 MAb to the surface antigens of Raji cell line was confirmed by both immobilizing methods, whereas this MAb had not a noticeable affinity to the MOLT-4 cells.

***Conclusion:*** According to the outcomes, the investigated MAb had acceptable affinity and specificity to the target antigens on the cell surface and could be utilized for immuno-detection of CD20-positive intact cells by SPR method.

## Introduction

Surface plasmon resonance (SPR) technology is widely used for the study of the interactions between a variety of chemical compounds and various biomolecules such as proteins, peptides and nucleic acids.^1^ Assessment of the interactions between analytes and immobilized ligands such as antibody/antigen and complementary nucleic acids is possible using this technology.^2,3^ Membrane proteins as important targets for drug discovery have recently attracted a great deal of interest for binding studies by this system.^4^ SPR based assessments are highly reproducible and permit the real-time investigation of probable interactions in label free form.^4^ CD20 is a surface protein which has been extensively utilized for targeted therapy of hematologic malignancies and autoimmune disorders.^5^ Rituximab was the first FDA approved anti-CD20 monoclonal antibody (MAb) for the targeted therapy of non-Hodgkin’s lymphoma and chronic lymphocytic leukemia.^6^ However, Rituximab and current therapeutics have not associated with complete remission in a considerable portion of the patients.^7^ Hence there is an urgent need for development and assessment of more efficient therapeutics. Targeted therapy, due to the reduced drug dosage and minimized harmful effects on unintended tissues has been considered as a more tolerable treatment approach.^8^ In addition to MAbs with native format, antibody derivatives have been also introduced for targeting studies.^5^ Bispecific antibodies (which simultaneously target two different antigens) and CAR T cells (engineered T cells with surface expression of chimeric antigen receptors) are examples of novel targeting agents.^9^ These agents acquire their antigen binding parts from the single-chain variable fragments (scFvs) of the input MAbs. Consequently, efficient binding of the utilized MAb is a prerequisite for engineering of this type therapeutics.

In the present study, the antigen-binding capacity of an anti-CD20 MAb was assessed by SPR system. Since proper functionalizing and assembly of the SPR chips is an important step to enable reliable detection of biomolecule binding,^1^ we optimized two distinct strategies for detection of a CD20-positive Burkitt's lymphoma cell line.

## Materials and Methods

N-hydroxysuccinimide (NHS), 11-mercaptoundecanoic acid (MUA), Bovine serum albumin (BSA) and PBS 10X were purchased from Sigma–Aldrich (Steinheim, Germany). Fetal bovine serum, penicillin and streptomycin were obtained from Gibco (Thermo Fisher Scientific, USA). Pure gold (Au) chips were acquired from bionavis company (Tampere, Finland). The recombinant *Staphylococcus aureus* protein A (SpA) was kindly provided by Dr. Gholamreza‏ Ahmadian and Dr. Garshasb‏ Rigi (Department of Molecular Genetics, National Institute of Genetic Engineering and Biotechnology, NIGEB).‏ The murine IgG2a anti-CD20 MAb was acquired from our previous works.^9,10^ Raji (a Burkitt's lymphoma cell line) and MOLT-4 (human T lymphoblast related to acute lymphoblastic leukemia) were purchased from the National Cell Bank of Iran (Pasteur Institute, Tehran, Iran).

### Cell culture

CD20-positive Raji cells and MOLT-4 cells as representative of CD20-negative T lymphoblasts were cultured in RPMI-1640 medium supplemented with 100U/ml penicillin, 100 μg/ml streptomycin and 10% fetal bovine serum and incubated at 37 °C in a humidified incubator with 5% CO_2_.

### Sensor surface cleaning 

SPR-Navi Au-slides are made of BK7- glass and coated with 50 nm of gold layer. For cleaning the Au surface, a solution composed of ammonia and hydrogen peroxide was used. In Brief, a solution containing 4 ml of ammonia (NH_4_OH), 4 ml of hydrogen peroxide (H_2_O_2_) and 12 ml Milli-Q-water was prepared in a Petri dish. Then, gold slides were immersed and boiled on a 95°C hotplate for 10 minutes. The slides were rinsed thoroughly with Milli-Q-water and dried with nitrogen stream.^11^

### Preparation of "protein A" chip for antibody immobilization 

**Figure 1:**
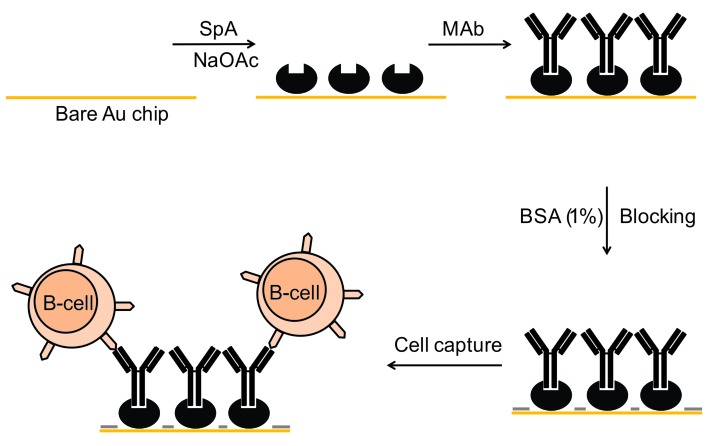
SpA mediated MAb immobilization on Au chip for cell detection

The *Staphylococcus aureus* protein A (SpA) contains immunoglobulin-binding domains which can efficiently attach to the Fc regions of a variety of IgG molecules such as murine IgG2a and human IgG1 subclasses.^12^ In the current study, a 0.5 mg per ml concentration of SpA was prepared in 10X PBS (pH7). 150 μl of SpA solution and 150 μl of Acetate buffer (800 mg of sodium acetate and 572 µl of acetic acid at pH 5.5) were coated on the Au surface of the cleaned chip.^13^ After 1h of incubation at 25°C, the chip was thoroughly washed with 1X PBS (pH 7) and dried with nitrogen stream. Subsequently, 200μl of anti-CD20 MAb (1mg/ml) was added on the surface and incubated for 1h. Afterward, 2ml of BSA (1% in 10X PBS) was passed through a millipore syringe filter with a pore size of 0.22 µm and 200µl of the filtered BSA was used for surface blocking. After 15 min of blocking, the chip was washed with PBS and utilized as cell binding platform (Figure 1).

### Preparation of MUA activated chip for antibody immobilization

**Figure 2:**
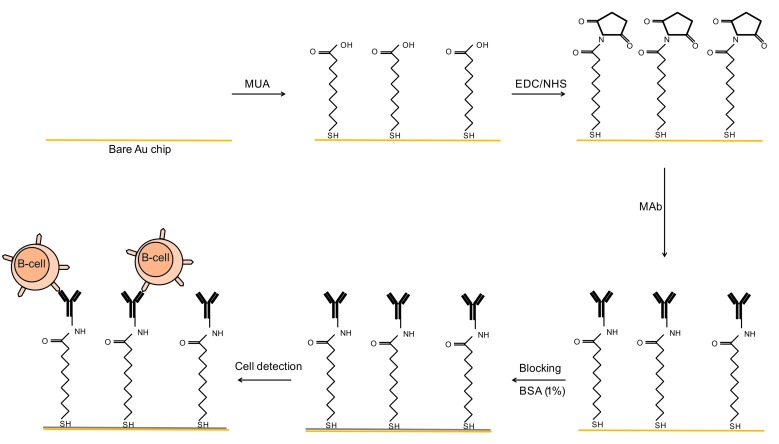
MAb immobilization on Au chip via MUA self assembled monolayer for cell detection

A 5mM concentration of MUA (in absolute ethanol) was used for creation of functional carboxyl groups for MAb binding.^14^ For this purpose, a bare Au slide was immersed in a solution containing MUA and Milli-Q-water in a ratio of 7:3. The slide was incubated at room temperature (~25°C) for 20 h and washed thrice with ethanol and then rinsed with phosphate buffered saline (PBS).^15^ The‏ functionalized gold slide was activated by NHS (0.05M) and EDC (0.2M). The Au chip was then treated with 200 μl of anti-CD20 MAb (1mg/ml). After 1‏ h incubation and complete washing/drying process, the chip surface was blocked‏ with 200 μ of filtered BSA (1% in 10X PBS). After 15 min, the chip was washed and used for immuno-sensing of the target cells (Figure 2).

### Cell capturing by the immobilized antibodies and SPR Measurements

A multi-parameter SPR device (MP-SPR Navi 210A, BioNavis Ltd, Tampere, Finland) was employed to investigate the antibody/cell interactions. This equipment utilizes the Kretscheman prism configuration with gold chips (BioNavis Ltd, Finland).^14^ Prior to cell injection, Raji and MOLT-4 cell were harvested from culture media. After counting with a hemocytometer, 5×10^4^ cells were washed and resuspended‏ in 1ml of PBS. All cell injections were accomplished at 30°C in PBS (pH 7.4)‏ as running buffer. Cell injection was performed in a 5 min period with a flow rate of 40 µl/min. After cell injection, the chips were rinsed with running buffer to remove the unbound cells. Data were analyzed using data viewer 210A SPR Bionavis software. In the current study, assessments were performed in fixed angle mode and a 670 nm laser was employed to excite the surface plasmon.

### Experiment normalization for non-specific binding

**Figure 3:**
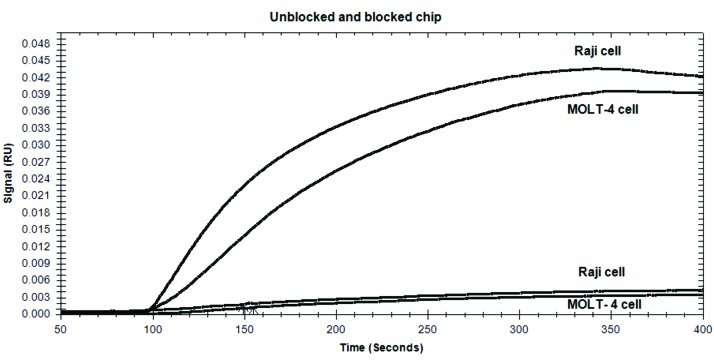
Sensorgrams of unblocked chip (above) and BSA blocked chip (below) for detection of nonspecific cell binding and selectivity of the biochip. Both Raji and MOLT-4 cells have bound to the unblocked chip, whereas their bindings to the BSA treated bare gold is negligible.

An unblocked Au chip was placed in the slide-holder of the SPR equipment. Suspended‏ Raji and MOLT-4 cells were injected via separate channels as mobile phase. The non-specific adhesion of the mentioned cells onto the bare Au surface‏ was detected and automatically‏ quantified (Figure 3). Using the same condition, a‏ BSA blocked chip (bare Au chip with no MAb immobilization but BSA blocked) was considered for the experiment normalization (Figure 3).

## Results

### Normalization of the non-specific binding 

Sensorgrams of BSA blocked chip and unblocked chip were acquired for detection of "selectivity of the biochip" and "nonspecific cell binding", respectively. As shown in Figure 3, the response unit values (RU) for unblocked chip for MOLT-4 and‏ Raji cells‏ were ~ 0.040 and ~ 0.043, respectively. Compared to the unblocked chip, the BSA blocked chip showed‏ very low RU values for nonspecific binding of MOLT-4 and Raji cells (~0.003)

### Immuno-detection by SpA mediated antibody immobilization method

**Figure 4:**
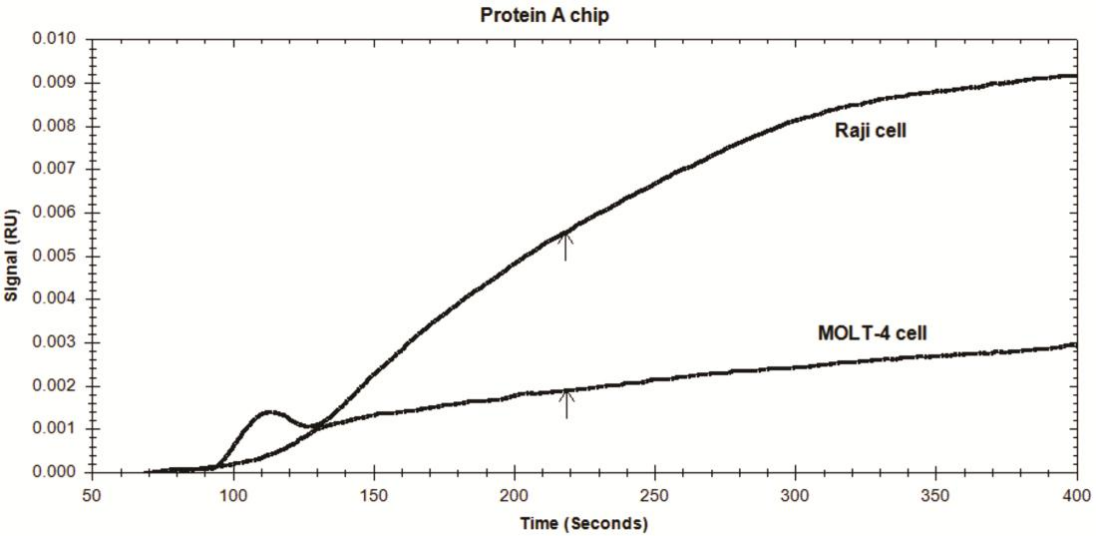
Sensorgrams of Raji and MOLT-4 cells detection by SpA immobilization method

Sensorgram‏ of SpA mediated approach showed RU values of 0.003 and 0.009 for MOLT-4 and Raji cells, respectively (Figure 4). Considering the measured value for the BSA blocked normalizer chip (~0.003) (Figure 3), MOLT-4 cells indicated a similar RU value. This means that, MOLT-4 cell line had no sensible binding to the SpA immobilized anti-CD20 MAbs,‏ whereas a time dependent increase in target binding was evident for CD20-positive Raji cells. However, the quantified value was significantly lower than the value measured for the unblocked bare Au chip (Figure 3). It could be deduced that, binding onto the SpA sensor chip has been specific.

### Immuno-sensing using MUA mediated antibody immobilization method

**Figure 5:**
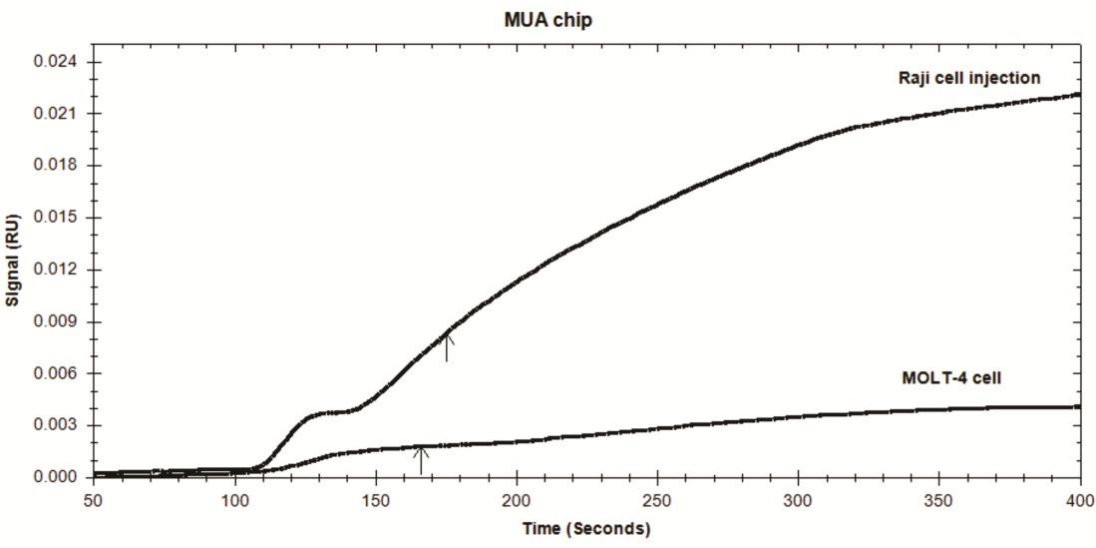
Sensorgrams of Raji and MOLT-4 cells detection by MUA mediated immobilization

Considering the corresponding sensorgram‏ (Figure 5), the binding value of CD20-negative MOLT-4 cells was ~ 0.003 RU which is equal to the negative (BSA blocked) normalizer chip. On the contrary, a time-dependent MAb/cell binding was measured‏ for Raji cells (~ 0.022).‏ This amount is significantly lower than the quantified value for the unblocked bare Au chip (Figure 3) indicating the selective binding of CD20-positive cells onto Au chip.

## Discussion

Several SPR-based experiments are designed for detection of isolated or recombinant antigens. However, detached antigens or recombinant proteins may fail to reflect the exact interactions of the *in vivo* interactions. CD20 is a surface protein which spans the cell membrane 4 times. Most of anti-CD20 MAbs bind to discontinuous epitopes on this molecule.^9^ It is expected that, the antigens on the cell surfaces which retain their natural three dimensional (3D) conformations, produce more factual binding values. In this regard, we tried to assess the interaction of a MAb with CD20 antigen on the intact cells. A number of studies have employed SPR for investigating ligand interactions with bacteria and mammalian intact cells.^16-19^ It should be noticed that, because of having evanescent filed near 400 nm on the Au surface, the size of the ligands to be immobilized is limited in SPR method.^18^ Alternatively, the interaction partner with applicable dimensions should be fixed on the sensor chip. In the present study, the antibody of interest was fixed on the Au chips and the investigated cells were injected as analyte.^1^ By running the apparatus, sensorgrams indicating the cell binding/detection rates were obtained for the two studied strategies. In the first approach, SpA was used for antibody fixation on Au chips. SpA, a surface protein of *Staphylococcus aureus,* is widely used for antibody purification in laboratory. This molecule has five Fc-binding domains and shows acceptable binding affinities to different immunoglobulins, especially human IgG1 and mouse IgG2a molecules.^12^

In the second method, MUA was used for the creation of a self-assembled monolayer on Au chip. The chemical functionalization of SPR chip with the aforementioned protocol resulted in the creation of free carboxyl groups on MUA molecules which in turn provided platforms for MAb immobilization.^20^‏

**Figure 6:**
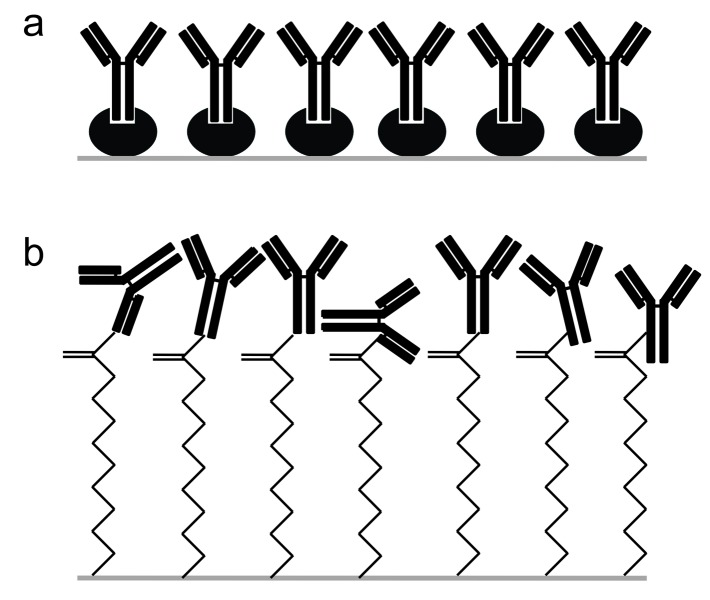
Schematic presentation of MAb immobilization approaches; a: oriented (with SpA) and b: random (with MUA)

MAbs are naturally composed of two main parts: The Fab region which is the fragment for antigen-binding, and the Fc region that is responsible for effector functions via binding to specific receptors on the cells of the immune system.^5^ Hence, accessibility of the Fab region is necessary for antigen binding. Since SpA binds to the Fc regions, the epitope binding sites of the MAbs are arranged in the opposite direction of the Au chip, available for antigen binding (Figure 6a). On the contrary, binding to amine groups of the MAbs in MUA method is completely random and some antigen binding sites become inaccessible (Figure 6b).^21^ Hence, due to the oriented fixation of the MAbs, the SpA mediated immobilization is expected to be more efficient.^13^ However, according to the results of this study MUA mediated immobilization demonstrated higher response units compared to the SpA method.

**Figure 7:**
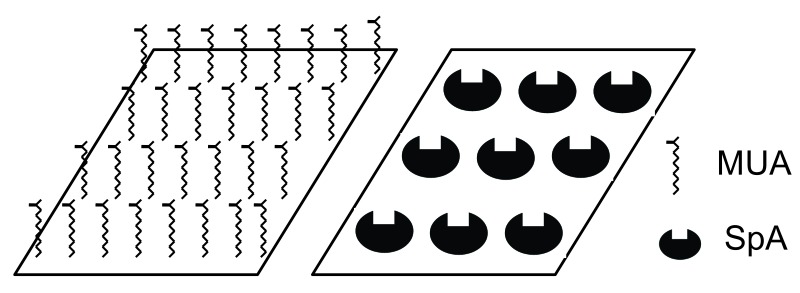
Hypothetic model for chip area occupation with SpA and MUA molecules

Hypothetically, MUA because of its filamentous structure may occupy smaller area compared to macromolecule SpA. Consequently, MUA can create greater density on the chip surface and this larger quantity compensate for the effects of irregular orientation (Figure 7).

Regardless of the strategy used, target specific interactions were evident in this study and nonspecific MAb/cell bindings were not reflected in the outcomes. According to the results of the both immobilization methods, the investigated antibody had an acceptable affinity and specificity to CD20 molecule.

## Conclusion

In this study, "11-mercaptoundecanoic acid" and *"Staphylococcus aureus* protein A" were utilized for immobilization of an anti-CD20 antibody on gold surface of SPR chips. According to the results, both strategies were applicable for this purpose and the created sensors were able to detect target cells. Furthermore, the investigated monoclonal antibody had acceptable binding specificity to CD20-positive Raji cells, whereas its interaction with CD20-negative MOLT-4 cells was negligible. Therefore, the introduced systems could be employed for immuno-detection of intact CD20-positive cells by SPR.

## Acknowledgments

This work was supported by a grant from Immunology Research Center, Tabriz University of Medical Sciences, Tabriz, Iran. Here, we wish to express our sincere gratitude and appreciation to Dr. Gholamreza Ahmadian and Dr. Garshasb Rigi for providing recombinant protein A.

## Ethical Issues

Not applicable.

## Conflict of Interest

The authors declare no conflict of interests.
